# An Account of the Practice of One of the Physicians of the Westminster General Dispensary, and of the Western Dispensary, from the 20th of March, to the 20th of April, 1808

**Published:** 1808-05

**Authors:** Samuel Fothergill

**Affiliations:** Leicester-Square


*An Account of the Practice of one of the Physicians of the Westminster General Dispensary, and of the Western Dispensary, from the 20th of March, to the 20th of April,* 1808.

ACUTE DISEASES.

| | |
|---|---|
| Catarrh | 8 |
| Synochus | 3 |
| Acute Rheumatism | 4 |
| Pleurisy | 2 |
| Peripneumony | 1 |
| Erysipelas | 3 |
| Hæmoptysis | 2 |
| Hooping Cough | 4 |
| Acute Diseases of Infants | 11 |

CHRONIC DISEASES.

| | |
|---|---|
| Cough and Dyspnœa | 25 |
| Asthma | 2 |
| Pulmonary Consumption | 6 |
| Debility and Cough after Measles | 4 |
| Pneumonia Notha | 5 |
| Pleurodyne | 2 |
| Chronic Rheumatism | 10 |
| Cephalalgia | 4 |
| Asthenia | 12 |
| Gastrodynia | 7 |
| Enterodynia | 3 |
| Colic | 2 |
| Dyspepsia | 5 |
| Constipation | 2 |
| Diarrhœa | 8 |
| Bilious Vomiting | 3 |
| Hæmatemesis | 2 |
| Worms | 3 |
| Dropsy | 5 |
| Scirrous Liver | 1 |
| Scirrus Uteri | 1 |
| Jaundice | 2 |
| Scurvy | 1 |
| Epilepsy | 1 |
| Palsy | 2 |
| Cutaneous Diseases | 6 |
| Chlorosis | 3 |
| Amenorrhœa | 2 |
| Menorrhagia | 1 |
| Leucorrhœa | 4 |

With the exception of two or three fine days at the beginning of this month, the weather has been uniformly severe; and if numbers of people are labouring under complaints induced by such undeviating and long protracted inclemency, we wonder less, than that so many still enjoy their usual state of health and flow of spirits.

The air which we constantly breathe, and which every where surrounds us, is liable to great variations of temperature and moisture, of rarity and of density; it is vitiated and rendered impure by suspending various noxious exhalations, vapours, and minute particles of matter; and being so immediately in contact with man, and essential to his existence, has a direct and extensive influence upon his constitution, both morally and physically. The question of suicide, or an energetic effort to remove the difficulties in which a man may be involved, is not unfrequently determined by the aspect of the sky. Some men of eminence, indeed, have denied that the atmosphere has any influence upon the human frame or mental faculties; and Dr. Johnson, son, in the loftiness of his contempt for such an opinion, triumphantly exclaims, "Surely, nothing is more reproachful to a being endowed with reason, than to resign its powers to the influence of the air, and live in dependence on the weather and the wind for the only blessings which nature has put into our power, tranquillity and benevolence. This distinction of seasons is produced only by imagination operating on luxury. To temperance, every day is bright; and every hour is propitious to diligence. He that shall resolutely excite his faculties, or exert his virtues, will soon make himself superior to the seasons; and may set at defiance the morning mist and the evening damp, the blasts of the east, and the clouds of the south." However we may admire this as a specimen of fine writing, and as a proof of the facility with which men of genius can support and adorn either side of an argument, we feel our infirmities too strongly to assent to its truth; and are reluctantly obliged to acknowledge, that he who can alike resist the influence of climate, the depressing effects of long continued gloomy skies and inclement weather, is possessed of powers which few men inherit or attain.

The diseases enumerated in the present Report are very similar to those mentioned in the last. No new case of measles has occurred to my notice; but many children who have had that complaint several weeks, and even months ago, still labour under debility and pulmonic affections, which are very difficult to remove, and not unfrequently terminate in consumption.

The observations which I made last month upon the use of arsenic in Chronic Rheumatism, have induced Mr. Jenkinson of Oxford, to favour me with some remarks upon the subject; and though his letter was not intended for public perusal, at my request he has kindly permitted me to insert part of it, which I have great satisfaction in doing, because the merits of the mineral solution, in Chronic Rheumatism, are not sufficiently known, and Mr. Jenkinson has long promoted its introduction into more general practice. He observes, that "The heat occasioned by it, (*arsenic*,) in some persons, about the fauces and in the stomach, if the dose be only moderate, will generally be obviated by taking the drops in a tea-cup full of liquid, and washing them down with as much more of the same vehicle, and the more bland and mucilaginous the liquid is the better; sometimes it is necessary to warm it a little. An equal number of drops of laudanum is the best guard against its effects effects in deranging the stomach and bowels.* It is sometimes necessary to intermit its use for a day or two, and administer a saline purgative by way of overcoming its determination to the intestines, which, according to my experience, oftener produces slight dysentery and tenesmus than common diarrhœa. I fear the medicine will never be so much employed as it deserves, from the apprehensions entertained about mineral remedies in general, and arsenic in particular; but I do not see how its frequent failure in so unmanageable a disease as Chronic Rheumatism is, in its protracted stages, will prevent the trial of it, since the best of the old remedies is still more liable to that objection: it certainly sometimes succeeds after all those you have mentioned have failed; and I believe medicine does not possess a single article amongst its specifics that succeeds universally, even in the proper cases for its administration.

"The arsenic does little good in recent cases, especially in young subjects; indeed, it can rarely be persevered in where the patient is not much reduced in strength, owing to the greatness of its stimulant power; for which reason it succeeds much better in old persons: but I agree with you in thinking that its efficacy is not *entirely* confined to the deep seated affections which Dr. Bardsley enumerates.

"Febrile symptoms, and Catarrh with hoarseness, and coryza particularly, are very apt to be excited by it in full and irritable habits; but I have alluded to these inconveniences arising from a *misuse* of the remedy in the Medical and Physical Journal for September, 1807. To them I will only now add, that arsenic will alleviate considerably in many cases of *nodosity* of long standing, and now and then go as far towards curing, as can reasonably be expected from the nature of that disease."

SAMUEL FOTHERGILL.

*Leicester-Square, April* 24, 1808.

